# Human Prostate Sphere-Forming Cells Represent a Subset of Basal Epithelial Cells Capable of Glandular Regeneration in Vivo

**DOI:** 10.1002/pros.21083

**Published:** 2009-11-24

**Authors:** Isla P Garraway, Wenyi Sun, Chau P Tran, Sven Perner, Bao Zhang, Andrew S Goldstein, Scott A Hahm, Maahum Haider, Christian S Head, Robert E Reiter, Mark A Rubin, Owen N Witte

**Affiliations:** 1 Department of Urology, David Geffen School of Medicine at UCLALos Angeles, California; 2 Jonsson Comprehensive Cancer CenterLos Angeles, California; 3 Department of Pathology, Weill Medical Center of Cornell UniversityNew York, New York; 4 Department of Microbiology, Immunology, and Molecular GeneticsUCLA, Los Angeles, California; 5 Division of Head and Neck Surgery, David Geffen School of Medicine at UCLALos Angeles, California; 6 Department of Pathology and Clinical Medicine, David Geffen School of Medicine at UCLALos Angeles, California; 7 Howard Hughes Medical Institute, Broad ISCBMLos Angeles, California

**Keywords:** prostasphere, prostate regeneration, prostate stem cell

## Abstract

**BACKGROUND:**

Prostate stem/progenitor cells function in glandular development and maintenance. They may be targets for tumor initiation, so characterization of these cells may have therapeutic implications. Cells from dissociated tissues that form spheres in vitro often represent stem/progenitor cells. A subset of human prostate cells that form prostaspheres were evaluated for self-renewal and tissue regeneration capability in the present study.

**METHODS:**

Prostaspheres were generated from 59 prostatectomy specimens. Lineage marker expression and TMPRSS-ERG status was determined via immunohistochemistry and fluorescence in situ hybridization (FISH). Subpopulations of prostate epithelial cells were isolated by cell sorting and interrogated for sphere-forming activity. Tissue regeneration potential was assessed by combining sphere-forming cells with rat urogenital sinus mesenchyme (rUGSM) subcutaneously in immunocompromised mice.

**RESULTS:**

Prostate tissue specimens were heterogeneous, containing both benign and malignant (Gleason 3–5) glands. TMPRSS-ERG fusion was found in approximately 70% of cancers examined. Prostaspheres developed from single cells at a variable rate (0.5–4%) and could be serially passaged. A basal phenotype (CD44+CD49f+CK5+p63+CK8−AR−PSA−) was observed among sphere-forming cells. Subpopulations of prostate cells expressing tumor-associated calcium signal transducer 2 (Trop2), CD44, and CD49f preferentially formed spheres. In vivo implantation of sphere-forming cells and rUGSM regenerated tubular structures containing discreet basal and luminal layers. The TMPRSS-ERG fusion was absent in prostaspheres derived from fusion-positive tumor tissue, suggesting a survival/growth advantage of benign prostate epithelial cells.

**CONCLUSION:**

Human prostate sphere-forming cells self-renew, have tissue regeneration capability, and represent a subpopulation of basal cells. Prostate 70: 491–501, 2010. © 2009 Wiley-Liss, Inc.

## INTRODUCTION

Adult multipotential stem cells (SCs) are responsible for the development, maintenance, and regeneration of the range of specialized cell types comprising mammalian tissues [1,2]. Self-renewal is a fundamental characteristic of SCs and refers to asymmetric divisions that give rise to genetically identical daughter cells in addition to more differentiated progenitors. Substantial evidence suggests that this process may be deregulated in cancer, as transformed SCs or early progenitors demonstrate uncontrolled self-renewal that results in phenotypically diverse tumors [3–5]. Isolation of these cells may allow antigenic/molecular profiling and the delineation of mechanisms that regulate self-renewal and differentiation [5,6].

Several studies have attempted to identify human prostate SCs [7–12]. Richardson et al. [10] found that the 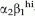-integrin cells co-expressing CD133 had increased proliferative activity, in vitro and limited tissue regeneration capacity, in vivo. The CD133+/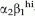 cells represent a small fraction (<1%) of prostate epithelial cells in benign and cancer tissues [8,10,12,13]. In a study of non-immortalized and immortalized human prostate cell lines, prostate stem cells were suggested to be 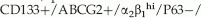
PSCA−/AR−/PSA− based on the low frequency of these cells in primary prostate epithelial (PrEC) cultures in relation to neuroendocrine and transient amplifying cells [12]. Additionally, sorted CD133+ cells yielded cultures containing a mix of CD133+ and CD133− prostate cells, representing the lineage cell types [11,12].

In order to adequately analyze prostate SCs and hierarchical order of prostate lineage cells derived from human tissue, methods that enable the efficient isolation and expansion of these rare cells is critical. Culture conditions that support proliferation of prostate cells that form spheres may represent a strategy for SC isolation [8,14–16]. Spheres are multicellular globes that develop from cells that survive anchorage-independent conditions in vitro, such as growth in ultra-low attachment (ULA) plates or 3D Matrigel cultures [17]. Spheres include SCs and early progenitors in studies of breast, brain, and skin and are frequently used to study the processes of self-renewal and differentiation in these systems [14,18–20]. Lang et al. [21] observed human sphere formation from dissociated prostate tissue. Spheroid formation and branching morphogenesis with expression of luminal markers in response to androgen and stromal growth factors was noted when primary human prostate cells were grown in 3D Matrigel cultures [21]. Other human studies have suggested that prostasphere formation is a functional validation of prostate SC activity in fractionated cell lines or xenografts [9,22,23]. In order to further characterize the relationship between stem/progenitor cells and sphere-formation, self-renewal and tissue regeneration properties of prostaspheres should be addressed.

In the present study, a diverse collection of human prostate surgical specimens was accrued and prostaspheres were generated reproducibly and robustly from all prostate tissue types when cells were cultured in low calcium, serum free, defined medium [12]. Clonally derived prostaspheres could be dissociated and passaged for multiple generations and induced the formation of ductal/acinar-like structures in vivo. Trop2, alpha 6-integrin (CD49f), and CD44 mark cells with enriched sphere-forming capability. The selfrenewing and differentiation features of prostaspheres support preservation of SC activity in these cultures. As previously suggested, low calcium, serum free culture conditions that support human prostate stem/progenitor growth appears to select exclusively for normal cells, since the *TMPRSS-ERG* fusion was never observed in the prostaspheres derived from fusion positive tissue specimens [11,12,24].

## MATERIALS ANDMETHODS

### Tissue Acquisition, Isolation, and Culture of Prostate Epithelial Cells

Human prostate tissue was obtained from 59 patients (ages 41–76), undergoing prostate surgery (radical prostatectomy or cystoprostatectomy). All subjects were consented for tissue collection in accordance with an approved protocol through the Office for the Protection of Research Subjects at UCLA. For a list of patient characteristics, see Supplemental Table I. Adjacent tissue specimens were fixed in formalin and paraffin-embedded to determine the presence of benign or malignant glands. The remainder of the tissue specimens were mechanically and enzymatically digested as previously described [25]. Dissociated prostate cell suspensions were sequentially filtered through 100- and 40-μm filters, and then passed through a 23-gauge needle. Cells were counted with a hemocytometer and resuspended in PREGM (Clonetics) supplemented with FGF2 (Invitrogen), EGF (Sigma), B27 (Invitrogen), and heparin (Sigma). The cells were then cultured (as described below), or subjected to cell separation using MACS beads columns (Miltenyi Biotec LTD, Surrey, UK) or a cell sorter, as described below.

### Antigenic Cell Separation

Cells were mixed with MACS microbeads linked to a cocktail of lineage antibodies (Lineage Depletion Kit, Miltenyi Biotec Ltd, Surrey, UK). After selection through the magnetic column, lineage negative cells were incubated with Trop-2 (R&D Systems), anti-CD44 (Abcam), anti-CD49f (Biolegend), or anti-CD133 (Miltenyi) antibodies, followed by incubation with MACS goat anti-mouse IgG microbeads (Miltenyi) and application to a MACS column. Alternatively, Lin— cells stained with fluorescent-linked primary antibody were subjected to sorting. Sorted cells were counted and plated in 3D Matrigel cultures.

### In Vitro Prostasphere Culture

Epithelial cells were counted and re-suspended in 50:50 matrigel:PREGM a concentration of 1 × 10^3^-6 × 10^4^cells/80 μl. This matrigel/cellular suspension was plated at the edge of the well on 12-well plates and allowed to set by incubation at 37°C for 30 min. One milliliter of defined media was then added to each well and plates were replaced in 37°C incubator. For dissociation and passage of prostaspheres, 1-hr incubation in 1 mg/ml Dispase (Invitrogen) was performed. Spheres were collected, washed in RPMI, and trypsinized (TripLE 100microliters/12-well plate). Cells were washed, counted, and replated as described above.

### Lentiviral Infection of Prostate Epithelial Cells

Prostate epithelial cells were cultured in PREGM for 48–72 hr. Viral supernatant containing lentivirus (CCR-dsRed, a gift from the laboratory of Dr. Irvin Chen at UCLA) and polybrene was added for 3 hr at 37°C. Red fluorescence was detected 48–72 hr postinfection. Monolayer cells were detached with TripLE and plated in Matrigel for sphere formation.

### Immunohistochemistry of Tissue/Prostasphere Sections

Prostate tissue was paraffin embedded as previously described [26]. For paraffin embedding of prostaspheres, matrigel cultures were subjected to Dispase (1 mg/ml, Invitrogen) and whole prostaspheres were collected and fixed in 10% buffered formalin at 4°C for 12 hr. After fixation, prostaspheres were washed in PBS and 50% ethanol, pelleted by centrifugation, and resuspended in 10-20 μl of Histogel (Richard-Allen Scientific). Four-micrometer thick sections of frozen or paraffin embedded tissue were deparaffinized with xylene and rehydrated through a descending series of ethanol washes as described. Antigen retrieval and standard immunoperoxidase procedures were used in combination with primary antibodies. For Immunofluorescence assays, permeablization of tissue was performed using cold methanol:acetone, followed by staining with antibodies.

### Fluorescence-Activated Cell Sorting and Analysis

Prostate cells were suspended in PBS/10% FCS and stained with antibody for 30 min at 4°C. Fluorescence-activated cell sorting and analysis are performed on a BD Special Order FACSAria II system and Diva v6.1.1. Live single cells are gated based on scatter properties and analyzed for their surface marker expression. Cells are sorted and collected at 40°C using 100 μm nozzle and 23 psi.

### Subcutaneous Injections in Immunocompromised Mice

Male SCID mice age 8–24 weeks was subjected to subcutaneous injections of prostaspheres ±2 × 10^5^ rat urogenital sinus mesenchyme (rUGSM) suspended in 100 μl 50:50 matrigel:PREGM. Subcutaneous implantation of time-release testosterone pellets was performed. Subcutaneous nodules at the site of injection were removed and frozen/paraffin-embedded sections were generated for immuno-histochemical analysis. Rat UGSM was prepared as previously described [27]. Fresh UGSM cells were cultured in DMEM + 10%FBS and passaged twice prior to use in tissue regeneration assays.

### Fluorescence Activated In Situ Hybridization

Paraffin-embedded human prostate tumor specimens were subjected to FISH as previously described [28]. Briefly, the break-apart assay is utilized with probes that recognize the centromeric and telomeric portions of the ERG gene. If there is a break in the gene, distinct red and green signals are detected. If the gene is intact, the red and green probes remain adjacent and sometimes overlap, resulting in a yellow signal (see Fig. 4).

## RESULTS

### A Small Fraction Human Prostate Cells Obtained From a Diverse Collection of Human ProstateTumor Specimens Form Prostaspheres

**Fig 1:**
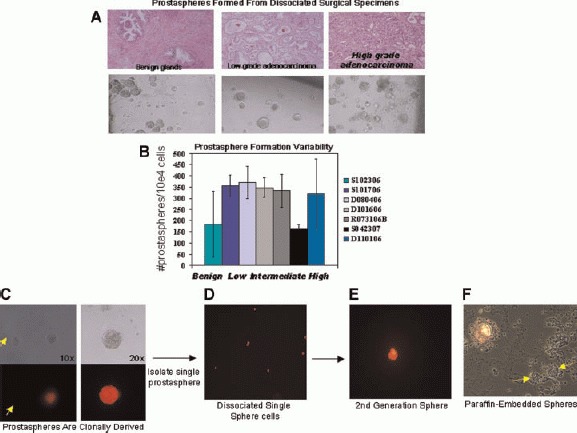
Formation of prostaspheres from dissociated human prostate tissue. Prostate tissue obtained from patients undergoing cancer surgery was mechanically and enzymatically digested. Single cells were and plated in Matrigel seeded at adensity 2 × 10^4^ /well in 6-well culture dishes. Adjacent tissue samples were paraffin-embedded and stained with H&E for histological evaluation. Human prostaspheres developed robustly from all prostate specimens (A), including benign prostate tissue, low-grade (Gleason 3 + 3) adenocarcinoma, and high-grade (Gleason 4 + 5) adenocarcinoma. Prostaspheres derived from these tissues formed prostaspheres in Matrigel (A, lower panel). B: Prostaspheres were plated in triplicate in 6-well plates at a seeding density of 1 × 10^4^ cells/well. After approximately 14 days, prostaspheres were counted and the average numberof spheres from seven individual patients with pathological diagnosis of benign prostate, low-grade, and high-grade adenocarcinoma is depicted in the graph. Freshly dissociated prostate cells were grown as a monolayer for 48 hr followed by incubation with lentivirus carrying the gene for red fluorescent protein (RFP). Red cells were mixed with uninfected (colorless) cells at a ratio of 1:5 and plated in Matrigel culture (1 × 10^4^ cells/well). Only monochromatic spheres were observed in (C) as seen in light and fluorescent views. Monochromatic spheres were isolated and plated by serial dilution into ULA96-well plates so that 1 -sphere/well was obtained. Single spheres were subjected to digest with TrypLE and single cells were then re-incubated in PREGM media (D). New monochromatic spheres were noted to form from single cells after 2 weeks of incubation (E). Frozen sections of RFP/clear spheres demonstrate that all cells within the sphere are red or clear and confirm clono-genicity (F).

To investigate whether sphere-forming cells may be derived from all types of human prostate tissue, specimens were obtained from 59 patients undergoing radical prostatectomy or cystoprostatectomy. Pathological examination of collected specimens confirmed inclusion of either benign (normal and BPH) or a mixture of benign and malignant glands with Gleason grades ranging from 3 to 5 (Supplemental Table I). Fresh tissues were mechanically and enzymatically dissociated and single cells were seeded in ULA plates or Matrigel at densities ranging from 10^2^ to 10^5^ cells/ well (Fig. 1, Supplemental Fig. 1). Prostaspheres formed from 21/24 specimens cultured in ULA plates and 35/35 specimens cultured in Matrigel with in 3 days of plating, with continued growth over 2 weeks to diameters of 100–400 μm (Fig. 1). There were only three patient specimens that failed to for prostaspheres, which may be attributed to variations in tissue processing as conditions were worked out. We observed that Matrigel cultures facilitated enumeration of prosta-spheres by preventing the aggregation of cells that occurs in floating culture [14,29]. Sphere architecture and marker expression was similar in both growth conditions (Supplemental Fig. 1).

Although significant variability in the number of prostaspheres that developed from individual patient specimens was observed, consistent variation in the number or appearance of prostaspheres according to specific clinical or pathological parameters was not apparent (Fig. 1A,B). Further prospective analysis with dissection/isolation of tumor nodules may allow more definitive conclusions on the relationship of sphere-formation and Gleason grade. Tallies of prostaspheres were obtained from a sample of 10 individual patients with varying pathologies (benign to malignant) after replicate plating in 6-well culture dishes. The average number of pros-taspheres obtained with similar seeding densities per patient was plotted (Fig. 1B). The frequency of sphere-forming cells derived from all 59 patients was found to range from approximately 0.5–4% (data not shown).

### Human Prostaspheres are Clonally Derived and Self-Renew

To confirm the clonal origin of prostaspheres, 10^5^ freshly isolated prostate epithelial cells were grown asa monolayer for 48 hr and then labeled with red fluorescent protein via lentiviral-mediated gene transfer of the dsRed gene. Approximately 90% of prostate cells appeared to be expressing dsRed within 72 hrafter exposure to virus (data not shown). Infected (red) prostate epithelial cells were detached and mixed with wild-type (colorless) cells at a ratio of 1:5. The mixed cell populations were plated in Matrigel in triplicate. Approximately 7 days following plating, robust prosta-sphere formation was observed and only monochromatic (all red or all colorless) prostaspheres were identified, consistent with the concept of clonality (Fig. 1C). Microscopic examination of frozen sections of DSRED and clear prostaspheres demonstrated that all cells within a single sphere were monochromatic (Fig. 1C–E).

All of the prostasphere specimens cultured in Matrigel could be passaged for multiple generations. Repetitive passaging was specifically assessed in four individual prostasphere cultures and more than 20 generations were obtained without any sign of growth decline after dissociation and passage (data not shown). DsRed-infected prostaspheres could also be dissociated, mixed, and passaged repeatedly (>3 generations) with formation of new monochromatic prostaspheres. Single red fluorescent prostaspheres were isolated by serial dilution in 96-well plates, followed by dissociation and incubation of single cells in Matrigel. Secondary red prostaspheres were noted to develop, supporting the self-renewal potential of individual prostaspheres (Fig. 1C–F).

Prostate cells obtained from dissociated prosta-spheres also remained viable after freeze/thaw, with formation of new spheres in Matrigel that could be serially passaged. Dissociated prostaspheres (passages 2–10) were cryopreserved for 1 month followed by thaw and seeding in Matrigel. New prostaspheres developed 7–10 days after plating that could be passaged >3 generations (data not shown). Taken together, these findings highlight the versatility of prostaspheres, in that they can be genetically manipulated, expanded continuously in vitro, and cryopre-served.

### Prostaspheres Express Predominately Basal Markers

The prostate epithelium is composed of basal cells, including stem cells and transient amplifying cells, terminally differentiated luminal cells, and neuroendocrine cells [30]. Prostate epithelial cells can be differentiated based on expression of a variety of markers [31]. The majority of basal cells express the high molecular weight cytokeratins (CK5 and CK14), p63, CD44, alpha integrin, and do not express significant low molecular weight cytokeratins (CK8/18), AR or PSA [12,25]. Luminal cells, in contrast, do not express p63, but exhibit relatively high levels of AR, PSA, CK8/ 18 [31]. Transient amplifying (intermediate) cells express basal marker, CK5 and often co-express the luminal marker, CK8 and prostate stem cell antigen (PSCA) [25]. Neuroendocrine cells are express neuropeptides, including chromogranin A and synaptophy-sin [31].

**Fig 2:**
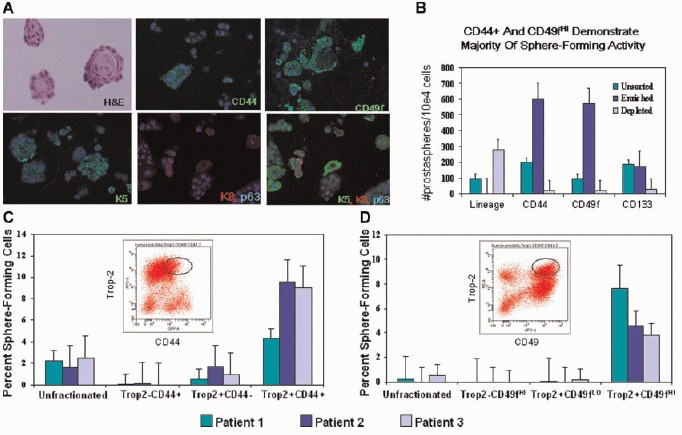
Human prostaspheres exhibit a predominately basal expression profile. Paraffin-embedded human prostaspheres were stained for prostate epithelial markers (A). H&Estaining, CD44 (green), CK5 (green), CK8 (red), and p63 (blue) are shown individually as well as co-staining. B. Fractionation of dissociated prostate cells via enrichment/depletion of lineage, CD44, CD49f, or CD133 antigens demonstrate sphere-forming capability of these cell populations. At least three different patient samples were tested per antigen. Lineage, CD44, and CD49f isolates were plated at a rate of 1 × 10^4^ cells per well. CD133 isolates were plated at 1 × 10^3^ cells/well. C,D: Percentage of spheres formed from prostate epithelial cells sorted based on Trop2/CD44 and Trop2/CD49f expression.

In order to determine the expression profile of prostate epithelial cells within prostaspheres, immu-nostaining was performed using antibodies against several basal and luminal markers (Fig. 2A). The basal markers CK5, alpha 6 integrin (CD49f), CD44, and p63 were strongly expressed by the majority of sphere-forming cells. Greater than 95% of the sphere-forming cells/20× objective high-powered field (hpf) appeared to express CK5 and p63. Between 50% and 80% of cells/ hpf expressed CD44 and CD49f. Luminal markers, including AR (androgen receptor) and PSA (prostate-specific antigen) were not observed in prostaspheres (data not shown). CK8 expression was noted in approximately 1% of sphere-forming cells/hpf. Co-expression with CK5 was seen in these cells (Fig. 2A). We did not observe cells expressing the neuroendocrine markers, synaptophysin or Chromogranin A (data not shown).

In addition to prostatic markers, the spheres were assessed for cell proliferation and apoptosis via Ki-67 and TUNEL staining (Supplemental Fig. 2). Approximately 2% of the sphere cells/hpf were observed to have Ki-67 activity, with the outermost layer of the sphere demonstrating the most activity. TUNEL staining was not clearly detected in prostaspheres (Supplemental Fig. 2).

### Antigenic Profile of Human Prostasphere-Forming Cells

To determine the surface markers that identify cells capable of prostasphere formation, we fractionated subpopulations of prostate cells via microbeads or automatic cell sorter separation (Fig. 2B–D). Cell fractionation resulted in a variable decrease (2–10-fold) in the overall number of prostaspheres formed following manipulation secondary to decreased cell viability following these manipulations (data not shown). However, comparison of fractionated cell populations within individual specimens allowed relative sphere-forming activity to be assessed.

In order to eliminate the possibility that cells of the hematopoeitic lineage retained in the prostate tissue contributed to the sphere-forming population, we performed depletions with blood lineage antibodies. FACS analysis with representative lineage antibodies CD31 and CD45 confirmed removal of hematopoeitic/ endothelial cells (Supplemental 3B). Lin— and Lin+ cells were plated in Matrigel cultures in several replicates of 1 × 10^4^cells/well to assess sphere-forming activity. Prostaspheres formed exclusively in the Lin-cell fraction (Fig. 2B).

Previous investigations of human and murine prostate SCs have suggested that CD44 may be an important marker [10]. In order to investigate whether CD44+ prostate epithelial cells were enriched for sphere-forming capability, we used microbeads or automatic cell sorting to isolate cells expressing this antigen. FACS analysis confirmed enrichment of CD44+ cells (Supplemental Fig. 3B). Plating of CD44+ cells in Matrigel demonstrated enriched sphere-forming capability (>2.5-fold) with 1/15 Lin—CD44+ cells forming spheres (Fig. 2B). Although the presence of CD44 greatly enriched for sphere-forming capability, some spheres were noted in the Lin—CD44— fraction. This likely represents incomplete separation of CD44+ and CD44— cells, but the possible that a fraction of Lin—CD44— cells have sphere-forming capability cannot be excluded.

We used a similar approach to examine whether CD49f or the putative human prostate stem cell marker, CD133, marked the sphere-forming population. FACS analysis demonstrated CD49f+ (8-20%) and CD133+ cells (2-7%) after lineage depletion (data not shown). Fractionated prostate cells were evaluated by FACS prior to seeding in Matrigel (Supplemental Fig. 3B). A marked increase in sphere formation was observed with Lin—CD49f + cells compared to Lin—CD49f — cells (Fig. 2B). On the other hand, multiple attempts to separate CD133+ and CD133— cells resulted in highly variable results in sphere-forming capability. For most of the patient samples, more spheres appeared to form in the CD133— fraction, however, a smaller number of spheres consistently formed within the CD133+ fraction as well (Fig. 2B). Our difficulties in obtaining consistent results with CD133 cell separation could be due to the fact that the CD133+ cells are so rare, usually composing 0.25-2% of unfractionated prostate cells. This made isolation and plating in sphere cultures difficult. We typically isolated <2 × 10^3^ CD133+ cells via this approach. Alternatively, CD133 as a solitary marker may subdivide, but not distinguish the sphere-forming population.

Our previous studies have demonstrated that human prostate sphere-forming cells are similar to murine prostate sphere-forming cells in regard to antigenic profile. The epithelial marker, Trop2 in combination with the high expression of the integrin, CD49f (a marker of human basal cells), enables sphere-forming cells to be isolated. Trop2 expression appears confined to epithelial cells in the human prostate and may be used in combination with other markers to evaluate subpopulations of prostate epithelial cells for sphere-forming capability. We performed cell sorting based on Trop2/CD49f and Trop2/ CD44 expression and found that the double positive fractions (Trop2+CD49fHI and Trop2+CD44+) demonstrate the highest sphere-forming capability in multiple patients (Fig. 2C-D).

### Prostaspheres Do Not Contain the *TMPRSS-ERG* Gene Rearrangement

Since the human prostaspheres were generated from primary tumors that contain a heterogeneous mix of benign and malignant glands, evaluation to distinguish benign and cancerous spheres, was warranted. However, qualitative and quantitative differences were not observed among prostaspheres derived from different pathological specimens (Fig. 1). With the discovery of prevalent gene rearrangements involving ETS family members in prostate cancer, we predicted that cytogenetic tools may enable identification of cancerous prostaspheres [28]. Gene fusions involving *ERG, ETV1,* and *ETV4* involve a variety of 5′ partners that direct aberrant expression of these transcription factors and may initiate a cascade of events leading to tumorigenesis [28]. The most common rearrangement involves juxtaposition of the androgen-regulated *TMPRSS2* gene with *ERG. TMPRSS-ERG* gene fusions have been detected in primary prostate tumor specimens, metastases, and xenografts by fluorescence in situ hybridization (FISH) [28]. Analysis of prostate tumor surgical cohorts have found 36-78% of prostate cancers possess the *TMPRSS-ERG* fusion [28]. The presence of this fusion in individual prostaspheres may suggest that cancer stem/early progenitor cells are expanded in our cultures.

**Fig 3:**
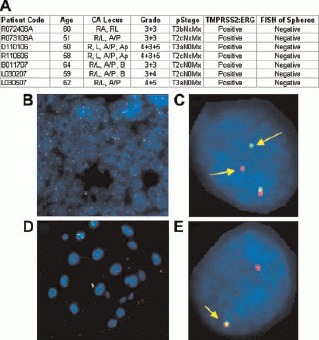
Patients undergoing radical prostatectomy for prostate cancer were consented for tissue donation according to an approved protocol through the Office for the Protection of Research Subjects at UCLA. Patient information including age, preoperative prostate specific antigen (PSA), tumor location on final pathology (R, Right, L, Left, A, Anterior, P, Posterior, Ap, Apex, B, Base), and pathological stage (T2, tumor confined within the prostatic capsule,T3, tumor extends beyond the prostate capsule) is presented in Panel A. Tissue specimens from these patients were mechanically and enzymatically dissociated and cultured in 50:50 matrigel:PREGM (supplemented with EGF, FGF, and B27) to allow prostasphere formation. Paraffin-embedded tumor specimens and prostaspheres were subjected to FISH for *TMPRSS-ERG* gene rearrangement. Human prostate cancer FISH is shown in Panels B (40×) and C (100×) in a case that displays the ERG break-apart genetic variation. In contrast, FISH of the prostaspheres shown in Panels D and E show an intact *ERG* gene with overlap of the probes. None of the prostaspheres derived from patients with *TMPRSS-ERG* fusion in their cancer specimens displayed a similar genetic abnormality.

To test the feasibility of this approach, FISH analysis was performed on select prostate tissue specimens and coordinating prostaspheres (Fig. 3). The *TMPRSS-ERG* fusion was found in approximately 7/10 (70%) cancer cases tested (Fig. 3A). The fusion, however, was consistently absent in prostasphere cultures derived from *TMPRSS-ERG*+ tissues, even when the specimens obtained contained >80% tumor (Fig. 3B-E). Analysis of monolayer cultures concomitantly derived from prostate tumor specimens also failed to demonstrate the presence of the gene fusion, indicating that both spheroid and adherent cultures select for fusion-negative, genetically normal cells (data not shown).

### Sphere-Mediated ProstateTissue Regeneration

**Fig 4:**
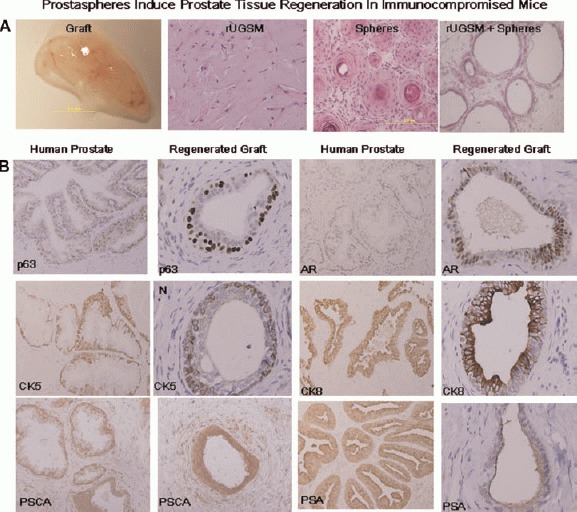
Prostaspheres form acinar structures in immunocompromised mice. Whole prostaspheres were isolated, mixed with Matrigel and injected subcutaneously into mice either with or without UGSM. Glandular-like structures were recovered 8–12 weeks after subcutaneous implantation of whole prostaspheres (containing from 5 × 10^4^ to 1 × 10^6^ cells) suspended in Matrigel. A: H&E staining of graft containing 2 × 10^5^ rat UGSM only, graft containing regenerated prostate tissue induced by prostaspheres without UGSM, and graft of regenerated prostate tissue formed by combining whole prostaspheres with 2 × 10^5^ rat UGSM. B: IHC analysis of the expression of CK5, p63, PSCA, AR,CK8, and PSA in normal human prostate tissue sections and acini induced by prostaspheres (regenerated graft).

To evaluate whether human prostaspheres can form ductal/acini structures in vivo, whole prostaspheres from eight patients were injected subcutaneously into NOD-SCID/IL2rγNull mice with or without 2 × 10^5^ rUGSM cells. The number of sphere forming cells injected ranged from 5 × 10^4^ to 1 × 10^6^. All experiments were performed in duplicate. In the mice injected with whole prostaspheres combined with Matrigel, 10/16 grafts were obtained 6-12 weeks post-injection (Fig. 4A). The grafts ranged in weight between approximately 25 and 250 mg. Control mice injected with rat UGSM without prostaspheres, were included for comparison (Fig. 4A). Grafts were fixed in formalin and embedded in paraffin to prepare tissue sections. H&E staining revealed the prostaspheres induced formation of acinar-like structures. No acini were observed in the rUGSM only grafts. In the absence of rUGSM, prostaspheres induced the development of rudimentary appearing acini/epithelial cords with multiple layers of epithelial cells and rare lumen formation (Fig. 4A). On the other hand, when human prostaspheres were combined with rat rUGSM and Matrigel, acini contained well-defined lumens with secretions (Fig. 4A). IHC analysis showed that these acinar structures were reminiscent of normal adult human adult prostate glands with CK8 and AR positive luminal cells as well as CK5 and p63 positive basal cells (Fig. 4B). Human prostate-specific markers, prostate stem cell antigen (PSCA), prostate membrane antigen (PSMA), and prostate specific antigen (PSA) were also observed (Fig. 4B). Immunostaining for the neuroendocrine markers synaptophysin and chromogranin A did not demonstrate neuroendocrine cells (data not shown). Although prostaspheres regenerated xenografts resembling normal human prostate, the efficiency of glandular structure formation was relatively low with 1-12 tubules ranging per 20× /hpf. Our results indicate that prostaspheres are capable of glandular regeneration with basal and luminal compartments comparable to normal human prostate tissue.

## Discussion

Sphere-formation in anchorage-independent conditions is a characteristic of SCs initially described in neural and mammary systems [16,18]. Studies of murine prostate have demonstrated that a small fraction of prostate epithelial cells expressing Sca-1 and alpha-6 integrin (CD49f), and Trop2 formed spheres in 3D-Matrigel cultures that possessed self-renewal and differentiation characteristics [14,17,21,32]. Additionally, only the sphere-forming fraction of murine prostate epithelial cells can induce gland formation via in vivo tissue regeneration [17,33]. In human studies, in vitro prostate spheroid formation with branching morphogenesis has been observed, but self-renewal, tissue regeneration capability, and antigenic profiling to delineate the sphere-forming population was not addressed [32,34]. Here, we show that 0.5–4% of epithelial cells obtained from a wide variety of dissociated human prostate specimens form prostaspheres that possess features of self-renewal, as demonstrated by serial passage. Human prostaspheres can also be dissociated and cryopreserved with the retention of sphere-forming ability following thaw (data not shown). These practical traits of human prostaspheres enable viable repositories of prostate stem/progenitor cells to be generated that could facilitate high throughput studies of large collections of patient specimens in the future.

In prior human prostate stem cell studies, epithelial cells expressing 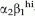 integrin, CD44, and CD133 have displayed stem-like qualities of increased proliferative potential in vitro and regeneration of acinar-like structures in vivo [7,10,13]. Consistent with previous reports characterizing prostate SCs, the putative SC markers, CD44 and α_6_ integrin (CD49f) appeared to greatly enhance for the sphere-forming population in this study, in addition to the epithelial marker, Trop2. The CD133+ population was technically difficult to evaluate, given the small fraction of these cells present in human prostate tissue specimens. Furthermore, recent studies suggest that CD133 antibody binding may inhibit survival of these cells in vitro [11]. It was observed that expression of CD133 did not segregate basal epithelial cells based on sphere-forming capability, since both CD133+ and CD133— fractions formed spheres. In contrast to CD44 and CD49f, immunostaining and FACS of prostaspheres failed to detect CD133, indicating that this marker is not preserved in prostasphere cultures (data not shown). The significance of CD133 expression should be evaluated in future studies examining characteristics of prostaspheres capable of tubule formation in vivo (see below).

The marker profile of sphere-forming cells with abundant expression of CK5 and p63 suggest that normal basal cells were selected in our prostasphere cultures. Basal markers are frequently lost in cancer and malignant glands display a luminal phenotype that includes abundant CK8/18 and AR expression, with loss of CK5 and p63 [35]. Until the discovery of ETS translocations, antigenic or genetic marker that clearly delineated normal and cancerous prostate cells were not available. With ETS family fusions now detectable via FISH, genetic events associated with malignancy can be evaluated in prostaspheres [28,36]. Consistent with the benign basal marker profile of prostaspheres and regenerated tubules, the *TMPRSS-ERG* fusion was not identified in sphere-forming cells from fusion+ tissue specimens. Although someofthe collected tissue specimens contained large tumor volumes (>80% tumor glands), all of the prostaspheres demonstrated a predominance of benign basal cells. Although it is possible that prostate cancer stem/progenitor cells do not contain gene rearrangements, a more likely scenario is that the culture conditions that support human prostate SC isolation and expansion inhibit cancer cell outgrowth. This observation is consistent with previous studies suggesting that cancer cells do not proliferate in the defined medium typically used to expand prostate epithelial cells [12,24]. Dissection of tumor nodules and outgrowth in more permissive media conditions may enable selection of prostate CSCs and will have to be addressed in future studies [24].

The acinar structures observedinvivo also appeared to resemble normal human prostate tissue, with preservation of discreet basal and luminal layers. In vivo tissue regeneration occurred at low efficiency with approximately 1 × 10 sphere forming cells yielding scant tubules in regenerated grafts. One possibility for the limited differentiation potential of prostaspheres is that a significant proportion of develop from progenitor cells, not bona fide SCs, and are not capable of tubule formation/maturation. To distinguish between human prostate SCs and progenitors, it will be necessary to further subdivide sphere-forming cells and evaluate tissue regeneration capacity of fractionated sphere-forming cells. It is possible that bona fide SCs will have the exclusive ability to recapitulate prostate glands in vivo, while progenitors will demonstrate restricted differentiation.

## CONCLUSIONS

Prostate sphere-forming cells include stem/progenitor cells that are capable of self-renewal and tissue regeneration. Sphere forming cells exhibit a basal profile that resembles benign prostate epithelial cells and induce the formation of ductal /acinar structures in vivo.
