# Supplementary figures and images for: Human Prostate Sphere-Forming Cells Represent a Subset of Basal Epithelial Cells Capable of Glandular Regeneration in Vivo
Source: Prostate. 2009 Nov 24;70(5):491–501. doi: 10.1002/pros.21083 (PMC2885946; doi:10.1002/pros.21083)

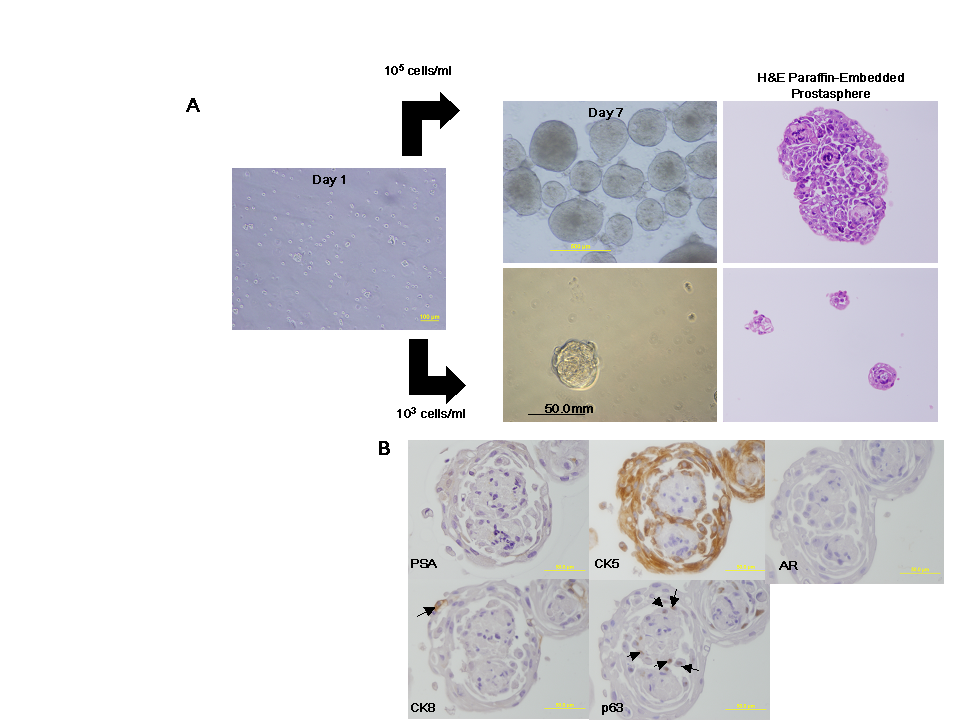

Supplement: Supplementary file 1 [file pros0070-0491-SD1.tif]

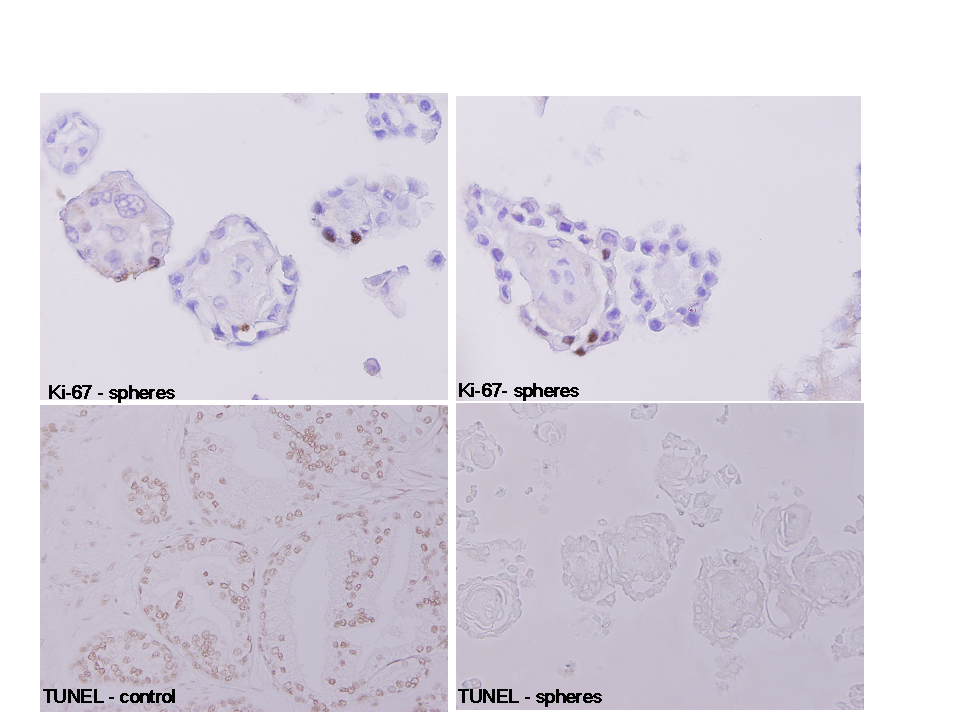

Supplement: Supplementary file 2 [file pros0070-0491-SD2.tif]

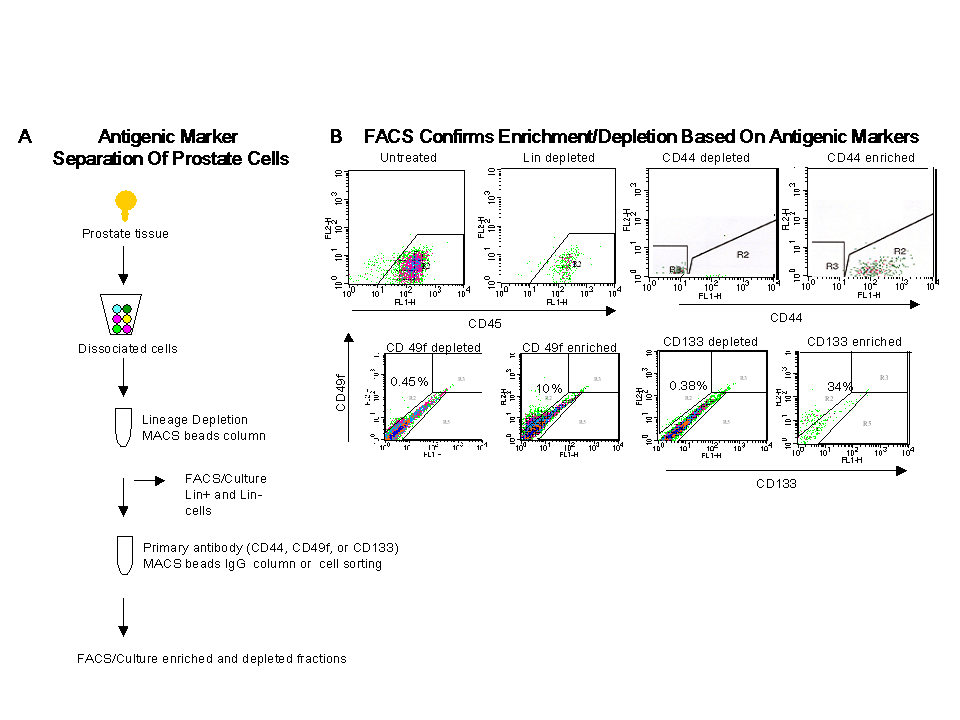

Supplement: Supplementary file 3 [file pros0070-0491-SD3.tif]

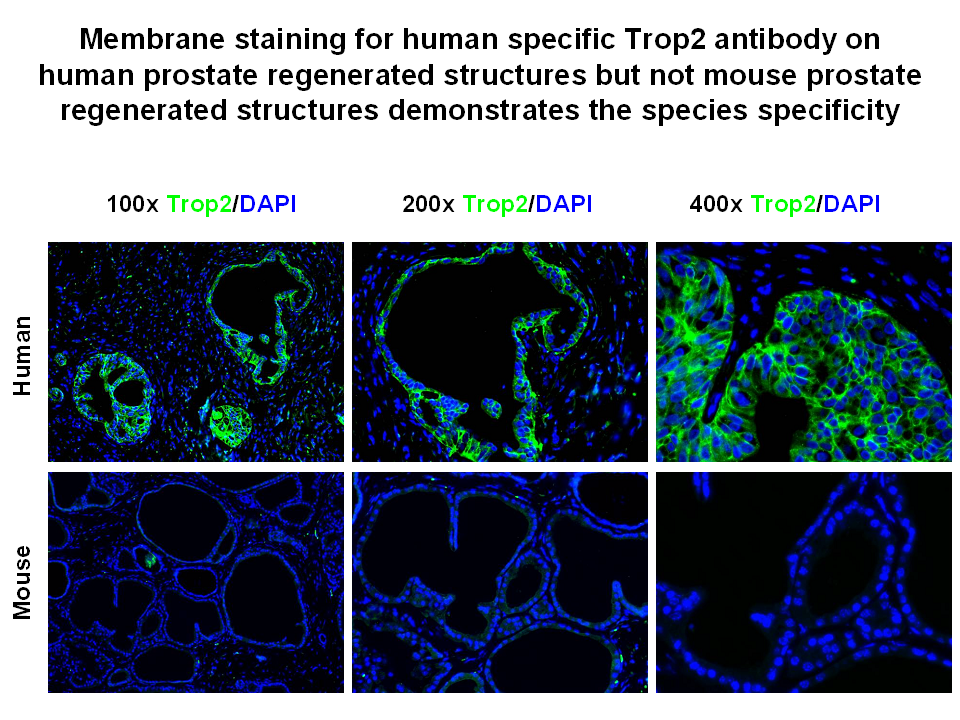

Supplement: Supplementary file 4 [file pros0070-0491-SD4.tif]
